# Evaluation of left ventricular strain in patients with arrhythmia based on the 3T MR temporal parallel acquisition technique

**DOI:** 10.1038/s41598-020-66315-z

**Published:** 2020-06-09

**Authors:** Hui Chen, Ru-Ming Xie, Lei Zhao, Xiao-Yong Zhang, Yi-Ke Zhao, Zheng Wang, Guo-Xi Xie, Xiao-Hai Ma

**Affiliations:** 1 Department of Radiology, Beijing Ditan Hospital, Capital Medical University, Beijing, 100015 China; 2 Department of Interventional Diagnosis and Treatment, Beijing Anzhen Hospital, Capital Medical University, Beijing, 100029 China; 3 Department of Radiology, Beijing Anzhen Hospital, Capital Medical University, Chaoyang District, Beijing 100029 China; 4 MR Collaborations NE Asia, Siemens Healthcare, Shenzhen, 518000 China; 5 Department of Biomedical Engineering, School of Basic Medical Science, Guangzhou Medical University, Guangzhou, 511416 China

**Keywords:** Cardiovascular diseases, Cardiology, Diseases, Cardiovascular diseases

## Abstract

Most of the current studies on myocardial strain are mainly applied in patients with sinus rhythm because the image quality of arrhythmias obtained with conventional scanning sequences does not meet diagnostic needs. Here, we intend to assess left ventricular (LV) global myocardial strain in patients with arrhythmias with 3 Tesla magnetic resonance (MR) and a new cine sequence. Thirty-three patients with arrhythmia and forty-eight subjects with sinus rhythm were enrolled in the study. LV myocardial thickness, cardiac function, myocardial strain and the apparent contrast-to-noise ratio (CNR) were all measured and compared using images generated by the real-time temporal parallel acquisition technique (TPAT) and the conventional cine sequence. In the arrhythmia group, the image quality of real-time TPAT was significantly better than that of the conventional cine sequence. In the arrhythmia group, the LV global peak radial strain and global peak circumferential strain values of real-time TPAT were significantly different from those of the conventional technique (radial strain, conventional: 20.27 ± 15.39 vs. TPAT: 24.14 ± 15.85, p = 0.007; circumferential strain, conventional:−12.06 ± 6.60 vs. TPAT: −13.71 ± 6.31, p = 0.015). There was no significant difference in global peak longitudinal strain between real-time TPAT and the conventional technique (−10.94 ± 4.66 vs. −10.70 ± 5.96, p = 0.771). There was no significant difference in the cardiac function parameters between the two techniques (p > 0.05), but there was a significant difference in 12 segments of the LV wall thickness between the two sequences (p < 0.05). In the sinus rhythm group, image quality using real-time TPAT was comparable to that using the conventional technique, and there was no significant difference in any of the indices (p > 0.05). Real-time TPAT is an effective method for detection of left ventricular myocardial deformation in patients with arrhythmia.

## Introduction

Over recent years, cardiac magnetic resonance (CMR) myocardial strain has developed rapidly and has been used for assessing myocardial deformation in many cardiovascular diseases, such as coronary artery disease and cardiomyopathy^1^. In particular, left ventricular (LV) global myocardial strain is superior to left ventricular ejection fraction (LVEF) for predicting cardiac adverse events^2^. LVEF is in the normal range in most arrhythmia patients without structural heart disease, but myocardial contractility is abnormal, which may lead to ventricular systolic dysfunction^3^ and may increase the risk of sudden death^4^. Therefore, it is important to evaluate myocardial deformation in patients with arrhythmia, and an evaluation index of ventricular wall contractility is needed.

CMR tissue tracking (CMR-TT) technology is widely used in patients with sinus rhythm, but it remains unclear whether it can be used in patients with arrhythmia. The large variation in heart rate can lead to motion artefacts in images acquired by conventional cine sequences with retrospective electrocardiogram (ECG)-triggering techniques (conventional techniques). With the rapid development of fast MR imaging techniques, real-time cardiac cine sequences have been gradually developed and applied in the clinic. In this case, the temporal parallel acquisition technique (TPAT) is used as a parallel imaging technique, which is based on the use of multi-channel coils for data acquisition and view-sharing schemes for image reconstruction. Compared with other real-time CMR methods based on the compressed sensing (CS) theory, the TPAT does not require nonlinear iterative methods or manual adjustment of penalty parameters for image reconstruction^5^. Therefore, the TPAT can achieve faster and more stable image reconstruction than real-time CMR imaging. However, it has not been used to assess LV myocardial strain in patients with arrhythmia.

In this prospective study, we sought to investigate the feasibility of the TPAT for evaluating LV myocardial strain in patients with sinus rhythm and then to assess LV myocardial strain in patients with arrhythmia.

## Results

In the arrhythmia group, 17 patients had atrial arrhythmias, and 16 patients had ventricular arrhythmias. Heart rate fluctuations were 3 to 90 beats/min (average 31.55 ± 22.72 beats/min), and heart rate variability was 10.55 ± 7.73% (Table 1).

**Table 1:** Clinical demographic data of the sinus rhythm group and arrhythmia group.

Groups	Sinus rhythm group (n = 48)	Arrhythmia group (n = 33)	p value
Male sex	34 (71%)	24 (73%)	0.853
Age (y)	51.02 ± 14.60	56.33 ± 11.08	0.161
Height (cm)	169.50 ± 8.18	168.88 ± 7.79	0.933
Weight (kg)	73.76 ± 9.60	69.61 ± 11.58	0.353
BMI (kg/m^2^)	25.64 ± 2.57	24.37 ± 3.42	0.145
BSA (m^2^)	1.83 ± 0.16	1.77 ± 0.18	0.492
Heart rate (beats/min)	73.52 ± 15.89	NA	
Average heart rate (beats/min)	NA	84.16 ± 15.22	
Heart rate variability (%)	NA	10.55 ± 7.00	
Hypertension	11 (24%)	13 (32%)	0.111
Diabetes mellitus	3 (9%)	5 (12%)	0.187
Dyslipidaemia	7 (15%)	8 (28%)	0.272
Coronary artery disease	7 (18%)	7 (20%)	0.438

BMI = body mass index, BSA = body surface area, NA = not available.

**Figure 1:**
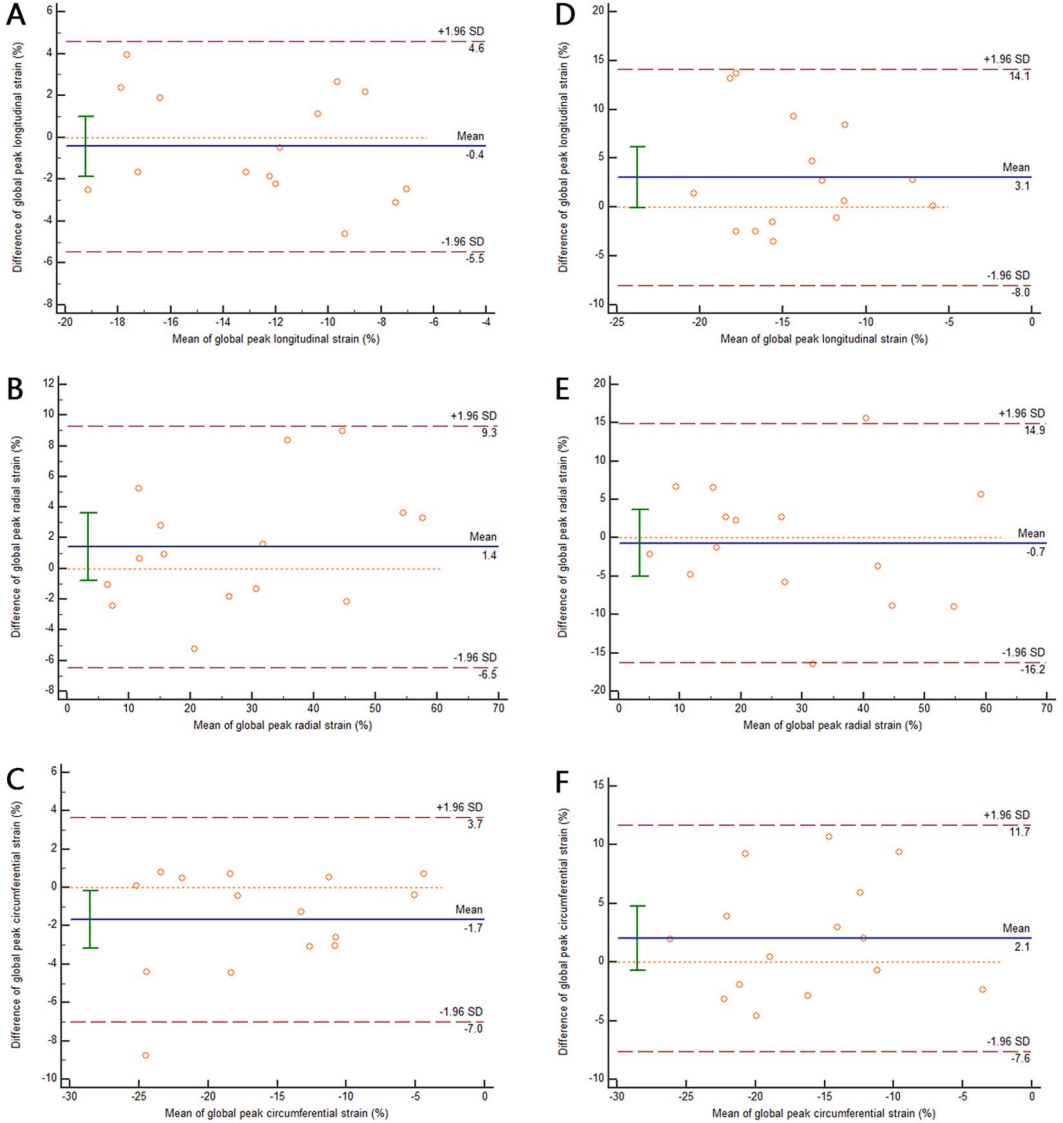
Bland-Altman plot of LV global peak longitudinal strain (**A**), radial strain (**B**) and circumferential strain (**C**) according to intra-observer reproducibility and of LV global peak longitudinal strain (**D**), radial strain (**E**) and circumferential strain (**F**) according to inter-observer reproducibility.

Images of fifteen subjects were randomly selected for intra- and inter-observer reproducibility, and circumferential, radial, and longitudinal strain analyses were performed independently by two experienced observers. Figure 1A–C are Bland-Altman plots for LV global peak longitudinal strain, radial strain and circumferential strain from intra-observers, and Fig. 1D–F are LV global peak longitudinal strain, radial strain and circumferential strain from inter-observers. As shown in Fig. 1B,C, global peak radial and circumferential strain had excellent agreement between measurements from intra-observers (global peak radial strain: intraclass correlation coefficient (ICC) = 0.972, 95% CI: 0.920~0.991; global peak circumferential strain: ICC = 0.927, 95% CI: 0.796~0.975), with minimal differences according to Bland-Altman analysis (global peak radial strain: 1.4 ± 4.0%, 95% CI: −6.5 to 9.3; global peak circumferential strain: −1.7 ± 2.7%, 95% CI: −7 to 3.7). For the measurements from two observers, global peak radial strain had excellent agreement (ICC = 0.895, 95% CI: 0.717~0.963), with a minimal difference of −0.7 ± 7.9% (Fig. 1E, 95% CI: −16.2 to 14.9).

**Figure 2:**
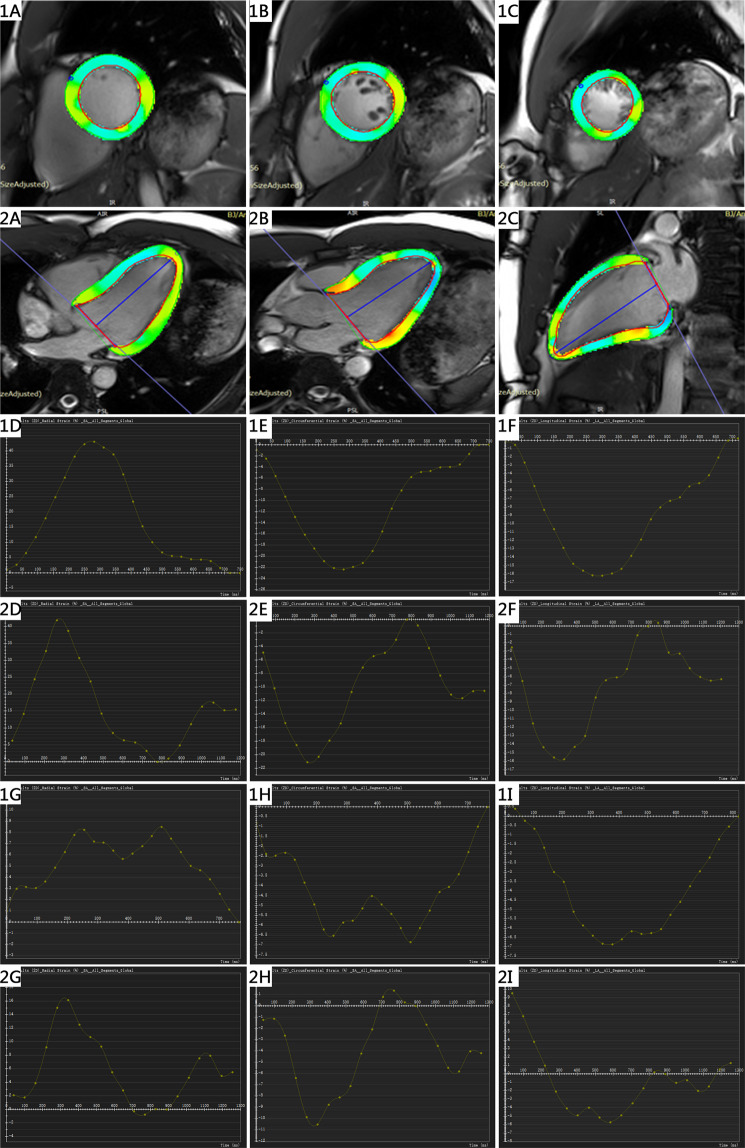
LV CMR tissue tracking. (**1A, 1B, 1C**) Radial strain and circumferential strain on the LV short-axis. Longitudinal strain on 4-chamber (**2A**), 3-chamber (**2B**) and 2-chamber (**C**) cine sequences. (**1D–1F, 2D–2F**) are the representative GRS, GCS and GLS curves from a sinus rhythm subject using the conventional and TPAT methods. (**1G–1I, 2G–2I**) are the representative GRS, GCS and GLS curves from an arrhythmia patient using the conventional technique and the TPAT.

In the arrhythmia group, the LV global peak radial strain and global peak circumferential strain values of the real-time TPAT were significantly different from those of the conventional technique (radial strain, conventional: 20.27 ± 15.39 vs. TPAT: 24.14 ± 15.85, p = 0.007; circumferential strain, conventional: −12.06 ± 6.60 vs. TPAT: −13.71 ± 6.31, p = 0.015). There was no significant difference in global peak longitudinal strain between the real-time TPAT and the conventional technique (−10.94 ± 4.66 vs. −10.70 ± 5.96, p = 0.771). There was no significant difference in the LV global peak radial strain, global peak circumferential strain or global peak longitudinal strain in the sinus rhythm group (p > 0.05, Table 2), although the strain indices derived from the conventional technique were higher than those derived from the real-time TPAT (Fig. 2).

**Table 2:** Comparison of LV peak global strain in both groups.

Parameter (%)	Arrhythmia group (n = 25)			Sinus rhythm group (n = 33)		
	Conventional technique	Real-time TPAT	P value	Conventional technique	Real-time TPAT	p value
Global peak radial strain	20.27 ± 15.39	24.14 ± 15.85	0.007	32.62 ± 14.27	30.65 ± 15.77	0.267
Global peak circumferential strain	−12.06 ± 6.60	−13.71 ± 6.31	0.015	−17.62 ± 5.85	−16.65 ± 6.20	0.187
Global peak longitudinal strain	−10.70 ± 5.96	−10.94 ± 4.66	0.771	−13.21 ± 6.34	−12.79 ± 7.09	0.763

Data are presented as the mean ± standard deviation.

**Figure 3:**
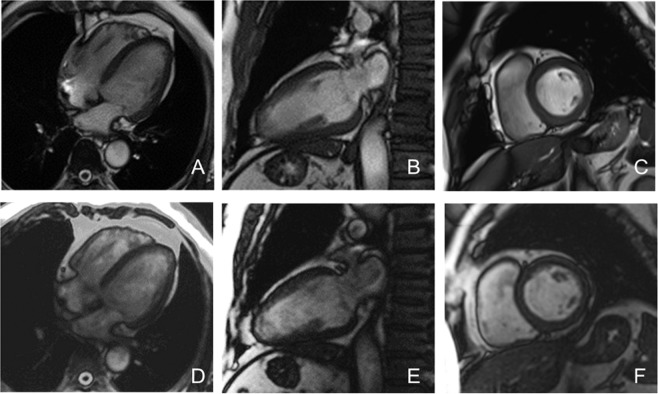
A 58-year-old male with sinus rhythm. **A~C** are the 4-chamber, 2-chamber and short-axis images of the conventional technique, and the image quality score was 4 points. **D~F** are the corresponding layers of the 4-chamber, 2-chamber and short-axis images of the real-time TPAT, and the image quality score was 4 points.

**Figure 4:**
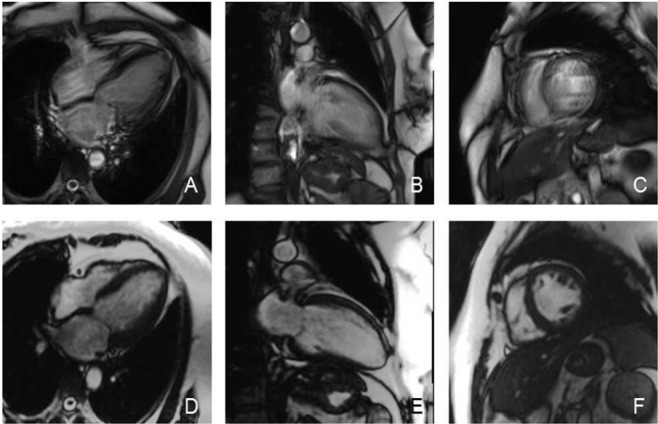
A 52-year-old male with arrhythmia. **A~C** are the 4-chamber, 2-chamber and short-axis images of the conventional technique, and the image quality score was 1 point. **D~F** are the corresponding layers of the 4-chamber, 2-chamber and short-axis images of the real-time TPAT, and the image quality score was 4 points.

In the arrhythmia group, the image quality of the real-time TPAT was significantly better than that of the conventional method (Z = 5.939, p < 0.001); however, in the sinus rhythm group, there was no significant difference in image quality between the two methods (Z = 0.050, p = 0.960) (Table 3). There was no significant difference in the contrast-to-noise ratio (CNR) between the two methods in both groups (p > 0.05) (Table 4; Figs. 3 and 4).

**Table 3:** The quality score of cine images in both groups.

Parameters	Arrhythmia group (n = 33)		Sinus rhythm group (n = 48)	
	Conventional technique	Real-time TPAT	Conventional technique	Real-time TPAT
4 points	1 (3.03)	16 (48.48)	28 (58.33)	28 (58.33)
3 points	6 (18.18)	15 (45.45)	16 (33.33)	17 (35.42)
2 points	18 (54.55)	2 (6.06)	4 (8.33)	1 (2.08)
1 point	8 (24.24)	0 (0)	0 (0)	2 (4.17)

Data are presented as n (%).

**Table 4:** Comparison of LV function parameters and CNR in both groups.

Parameters	Arrhythmia group (n = 33)			Sinus rhythm group (n = 48)		
	Conventional technique	Real-time TPAT	p value	Conventional technique	Real-time TPAT	p value
LVEF (%)	31.19 ± 15.84	32.62 ± 18.13	0.334	45.15 ± 17.78	45.03 ± 17.49	0.730
LVEDV (ml/m^2^)	83.85 ± 56.24	86.12 ± 53.94	0.117	76.36 ± 32.62	76.00 ± 32.39	0.572
LVESV (ml/m^2^)	56.47 ± 53.05	70.48 ± 62.02	0.138	45.85 ± 33.27	45.81 ± 33.15	0.911
SV (ml/m^2^)	21.48 ± 9.46	22.95 ± 10.07	0.160	29.88 ± 11.91	30.20 ± 10.22	0.666
CO (L/min)	3.01 ± 1.38	3.25 ± 1.57	0.109	4.13 ± 1.81	4.05 ± 1.65	0.248
MM (g/m^2^)	65.09 ± 32.94	63.25 ± 33.39	0.602	58.12 ± 24.31	57.78 ± 27.81	0.826
CNR	−5.86 ± 1.48	−6.10 ± 1.85	0.526	−6.11 ± 1.52	−7.23 ± 5.0	0.110

Data are presented as the mean ± standard deviation.

LV = left ventricle, CNR = contrast-to-noise ratio, EF = ejection fraction, EDV = end-diastolic volume, ESV = end-systolic volume, SV = stroke volume, CO = cardiac output, MM = myocardial mass.

In the sinus rhythm group, there was no significant difference in the 16 segments of LV myocardial thickness between the real-time TPAT and the conventional technique (p > 0.05). However, in the arrhythmia group, except for 4 segments [the inferoseptal and inferolateral segments of the LV basal level and the anterior and lateral segments of the LV apical level], there were significant differences in the remaining 12 segments (p < 0.05) (Table 5).

**Table 5:** 16-segment LV myocardial thickness for the arrhythmia and sinus rhythm group.

Segments	LV myocardial thickness (cm) in the arrhythmia group (n = 33)			LV myocardial thickness (cm) in the sinus rhythm group (n = 48)		
	Conventional technique	Real-time TPAT	p value	Conventional technique	Real-time TPAT	p value
Basal						
1 anterior	0.7 ± 0.2	0.7 ± 0.3	0.000	0.7 ± 0.2	0.7 ± 0.2	0.552
2 anteroseptal	1.1 ± 0.3	1.0 ± 0.3	0.000	1.1 ± 0.3	1.1 ± 0.3	0.069
3 inferoseptal	1.1 ± 0.3	1.0 ± 0.3	0.328	1.0 ± 0.3	1.0 ± 0.3	0.059
4 inferior	0.7 ± 0.2	0.7 ± 0.2	0.007	0.7 ± 0.2	0.7 ± 0.2	0.224
5 inferolateral	0.8 ± 0.2	0.8 ± 0.2	0.323	0.8 ± 0.2	0.7 ± 0.2	0.119
6 anterolateral	0.8 ± 0.2	0.7 ± 0.2	0.014	0.8 ± 0.2	0.7 ± 0.2	0.265
Middle						
7 anterior	0.7 ± 0.2	0.6 ± 0.2	0.014	0.7 ± 0.2	0.7 ± 0.2	0.910
8 anteroseptal	1.0 ± 0.4	0.9 ± 0.4	0.010	0.9 ± 0.3	0.9 ± 0.3	0.408
9 inferoseptal	1.1 ± 0.4	1.1 ± 0.4	0.008	1.0 ± 0.4	1.0 ± 0.4	0.056
10 inferior	0.7 ± 0.2	0.6 ± 0.2	0.000	0.7 ± 0.2	0.7 ± 0.2	0.855
11 inferolateral	0.8 ± 0.2	0.7 ± 0.2	0.007	0.7 ± 0.2	0.7 ± 0.2	0.936
12 anterolateral	0.8 ± 0.2	0.7 ± 0.2	0.014	0.7 ± 0.2	0.7 ± 0.2	0.082
Apical						
13 anterior	0.7 ± 0.2	0.6 ± 0.2	0.066	0.6 ± 0.2	0.6 ± 0.2	0.118
14 septal	0.6 ± 0.3	0.9 ± 0.3	0.03	0.9 ± 0.3	0.9 ± 0.3	0.353
15 inferior	0.7 ± 0.2	0.6 ± 0.2	0.014	0.6 ± 0.2	0.6 ± 0.2	0.482
16 lateral	0.7 ± 0.3	0.7 ± 0.3	0.116	0.6 ± 0.2	0.6 ± 0.1	0.242

There was no significant difference in the cardiac function indices between the two groups (p > 0.05) (Table 4).

## Discussion

Myocardial strain is closely related to the anatomical structure of heart muscle fibres^6^. CMR-TT imaging can be used to analyse myocardial motion and torsion^7,8^. The advantage of this approach is that it can quantitatively detect subtle myocardial deformation in coronary artery disease, cardiomyopathy, heart failure and other cardiac diseases^9–11^. Moreover, it can be measured on a cine sequence without special sequence scanning, and many studies have shown that CMR-TT technology has good repeatability, accuracy, and stability^12,13^. Strain is a basic parameter of CMR-TT. It changes continuously throughout the cardiac cycle, and myocardial deformation occurs in three directions^14^. Negative strain values represent myocardial circumferential and longitudinal shortening, while positive strain values represent radial thickening^15^. CMR studies have sparsely reported strain performance in patients with arrhythmia. In this study, the conventional technique and the real-time TPAT were used to explore LV myocardial strain and its characteristics in patients with arrhythmia.

This study showed that there was no significant difference in left ventricular global peak longitudinal, circumferential or radial strain as well as cardiac functional indices and myocardial thickness parameters between the real-time TPAT and the conventional technique in the sinus rhythm group, which indicated that the real-time TPAT had good performance feasibility. After testing the feasibility in the sinus group, we tried this sequence in arrhythmia patients. In the arrhythmia group, the image quality was improved with the TPAT compared to with the conventional sequence. Furthermore, there were significant differences in assessing the LV global peak radial strain and global peak circumferential strain values between the TPAT and the conventional technique, but there was no significant difference in the global peak longitudinal strain. Bilchick *et al*. used displacement encoding with stimulated echoes (DENSE) strain to assess myocardial deformation in 100 patients after cardiac resynchronization therapy. DENSE is a rapid imaging technology. Myocardial strain was analysed using cine images obtained from DENSE technology, and the results showed that the combined use of strain imaging and clinical risk models represented a promising paradigm to predict patients' risk of arrhythmia^16^. In the present study, we applied the real-time TPAT, which is also a rapid imaging technique, and found that patients with arrhythmia had impaired strain indices. Heermann *et al*. analysed the LV strain characteristics of 16 patients with arrhythmogenic right ventricular cardiomyopathy (ARVC) using CMR feature tracking technology. The absolute values of LV global longitudinal strain and radial strain of ARVC were lower than the normal reference values^17,18^. Moreover, Orwat *et al*. enrolled 372 patients after repair of tetralogy of Fallot and analysed the longitudinal strain, circumferential strain and radial strain using the CMR feature tracking method. The results showed that global longitudinal strain, circumferential strain and radial strain were all impaired and were associated with the New York Heart Association (NYHA) class and symptomatic deterioration^19^. In our study, the arrhythmia group included 17 patients with atrial arrhythmias and 16 patients with ventricular arrhythmias, and the global peak longitudinal strain, circumferential strain and radial strain in these patients were also lower than the normal reference values.

In the arrhythmia group, there were significant differences in 12 LV myocardial segments of the wall thickness between the two techniques. This result indicates that LV wall motion artefacts in patients with arrhythmia can affect the evaluation of heart structure with the conventional technique. In the arrhythmia group, there was no significant difference in the cardiac function parameters between the two technologies, which may be due to the real-time TPAT technology being as suitable as the conventional technique for capturing EDV and ESV in the cardiac cycle. To a certain extent, the real-time TPAT can be applied for the measurement of LV peak strain. However, in the condition of patients with fast ventricular rate, the temporal resolution of real-time TPAT maybe not enough for capturing EDV and ESV accurately. Further study is needed to clarify the suitable range of ventricular rate for real-time TPAT technique.

In recent years, compressed sensing for real-time cardiac cine sequences has also been developed^20,21^. Bassett *et al*. evaluated the performance of eight-fold-accelerated cine MRI sequences using compressed sensing in animals with tachycardia at 3 T, revealing that eight-fold real-time cine MRI sequences with k–t SPARSE-SENSE can achieve acceptable diagnostic quality and relatively accurate LV functional measurements of tachycardia^22^. However, image reconstruction of compressed sensing requires nonlinear iterative reconstruction and manual adjustment of the penalty parameter to balance data consistency and sparse constraints.

The current study has some limitations. First, the sample size was relatively small. Second, the cardiac morphology and functional parameters of patients with different arrhythmias were not further analysed. Third, compared with that of the conventional sequence, the spatial and temporal resolution of the real-time TPAT sequence was inferior, but for patients with arrhythmias, it could significantly improve image quality.

For sinus rhythm patients, we compared the real-time TPAT with conventional cine sequences, and the results showed that the TPAT could obtain similar image quality to conventional cine sequences. Therefore, the real-time TPAT can be used as an alternative sequence in patients with sinus rhythm. For patients with arrhythmia, the real-time TPAT can improve the image quality, and it is a scan sequence suitable for these patients to detect left ventricular myocardial deformation.

## Methods

### Ethical statement

The study was approved by the ethics committee of Beijing Anzhen Hospital, Capital Medical University, and the number was 2016031×. The study was carried out under relevant guidelines and regulations (Declaration of Helsinki). All participants gave written informed consent.

### Study population

The study included 81 patients (58 males, mean age 53.19 ± 13.46 years) who underwent CMR examination. Their indications for CMR included arrhythmia, myocardial infarction, hypertension, myocardial disease, heart failure, and myocarditis. According to the cardiac rhythm status during the examination, the patients were classified into the arrhythmia group (n = 33) or the sinus rhythm group (n = 48). In the arrhythmia group, ventricular premature beats, atrial fibrillation, and bigeminal beats were found, while in the sinus rhythm group, the cardiac rhythm was stable, and the R-R interval was equal.

### MR imaging

All CMR scans were performed on a 3 T MR system (MAGNETOM Verio, Siemens Healthcare, Erlangen, Germany) with a 32-channel cardiac coil. The patients were trained to hold their breath before scanning. During scanning, patients were scanned by conventional cardiac cine sequences with retrospective ECG-triggering (conventional technique) and real-time cardiac cine sequences with the TPAT (real-time TPAT). Both sequences were based on balanced steady-state free precession (bSSFP) readouts to obtain a better image signal-to-noise ratio (SNR) and contrast-to-noise ratio (CNR) between the myocardium and blood pool. Short-axis, four-chamber, three-chamber and two-chamber views of LV were acquired. The parameters of the TPAT were optimized to realize real-time cardiac imaging. The optimization included (1) using a lower spatial resolution (2.9 mm × 2.1 mm) and making the bandwidth (1359 Hz/pixel) as high as possible to shorten the echo spacing and thus improve the temporal resolution and to reduce banding artefacts; (2) setting the acquisition window equal to cardiac phases × temporal resolution to realize real-time cardiac imaging; and (3) optimizing the acceleration factor of the TPAT by balancing the temporal resolution and signal-to-noise ratio. The scanning parameters of the conventional technique and the real-time TPAT are shown in Table 6. The heart rate during scanning of every image was recorded; heart rate variability was calculated as the standard deviation from the average heart rate.

**Table 6:** Summary of sequence parameters.

Parameters	Conventional technique	Real-time TPAT
FOV (mm^2^)	340 × 289	340 × 289
TR (ms)	3.50	2.50
TE (ms)	1.51	1.12
Scan time per slice (s)	12	2.6
Temporal resolution (ms)	41.10	60.48
TPAT factor	—	4
Voxels (mm^3^)	1.3 × 1.3 × 8.0	2.9 × 2.1 × 8.0

### Image analysis

All cine images were transferred to an off-line workstation (Viewing and Argus, Siemens Healthcare, Erlangen, Germany) for analysis.

### Evaluation of left ventricular strain

Global LV peak strain analyses were performed using post-processing software (Tissue Tracking, CVI42, Circle Cardiovascular Imaging, Calgary, Canada). At end-diastole, endocardial and epicardial boundaries were manually delineated in 2-, 3-, 4-chamber and short-axis views, and papillary muscles were included within the endocardial borders. In the short-axis orientation, the insertion points of the right ventricle were manually delineated. Then, in 2-, 3-, and 4-chamber view images, straight lines were made at the mitral annular level and from the midpoint of the mitral annulus to the apex. After the automated tracking algorithm was applied, automatically rendered contours were reviewed and corrected. The analysis was performed over the entire cardiac cycle, resulting in a strain-time curve (Fig. 2). Because of mismatches in the long-axis scan plane, a total of 58 patients’ strain data were measured, and the remaining 23 patients’ images were not analysed.

### Evaluation of left ventricular myocardial thickness

Quantitative myocardial thickness analysis was performed according to the American Heart Association (AHA) segmental model for 16 segments (except the apical segment) in short-axis orientation^23^. In cine images of the real-time TPAT and the conventional technique, at the end-diastolic phase, the myocardial thickness of 16 segments of the LV was measured on the LV basal, middle and apical levels of the short-axis view images.

### Evaluation of global left ventricular function

Global left ventricular function analysis was performed using Siemens Syngo Argus post-processing software. LV endocardial and epicardial borders were manually traced in both the end-diastolic and end-systolic phases in the short-axis view. To reduce observer variability, LV papillary muscles were included as part of the LV end-diastolic volume and end-systolic volume and were excluded from the LV mass. Parameters of LV function were calculated by an automated algorithm, including LVEF, LV end-diastolic volume (LVEDV), LV end-systolic volume (LVESV), stroke volume (SV), cardiac output (CO) and myocardial mass (MM). To obtain standardized parameters, the above parameters except LVEF were adjusted according to body surface area.

### Evaluation of image quality

The overall image quality of each cine sequence was assessed using a four-point scale (4, excellent quality, no artefact; 3, good quality, slight artefact; 2, moderate artefacts; and 1, severe artefacts, unable to evaluate). All the analyses were performed by two independent radiologists on an off-line workstation, and if the two ratings were inconsistent, then a consensus was reached through discussion.

CNR was measured by one radiologist. The sizes of the region of interest (ROI) in the blood pool and myocardial structure were contoured on the images obtained by the conventional technique according to the size of the heart at the LV basal, middle and apical levels. Next, the ROIs were copied to images acquired by the real-time TPAT. Since the spatial resolution was different between the two techniques, it was necessary to manually adjust the copied ROIs to ensure that the contoured ROIs on the images of the two techniques were similar. The signal intensity and standard deviation of the blood pool and myocardium were measured and expressed as S _blood_, S _Myocardial_, SD _blood_, and SD _Myocardial,_ respectively. The CNR between the blood pool and myocardium was also calculated according to a previous work^24^ and was formulated as the following equation (1):

$${\rm{CNR}}=\frac{{\rm{M}}{\rm{E}}{\rm{A}}{\rm{N}}({{\rm{S}}}_{{\rm{M}}{\rm{y}}{\rm{o}}{\rm{c}}{\rm{a}}{\rm{r}}{\rm{d}}{\rm{i}}{\rm{a}}{\rm{l}}})-{\rm{M}}{\rm{E}}{\rm{A}}{\rm{N}}({{\rm{S}}}_{{\rm{B}}{\rm{l}}{\rm{o}}{\rm{o}}{\rm{d}}})}{\sqrt{{({\rm{S}}{{\rm{D}}}_{{\rm{M}}{\rm{y}}{\rm{o}}{\rm{c}}{\rm{a}}{\rm{r}}{\rm{d}}{\rm{i}}{\rm{a}}{\rm{l}}})}^{2}+{({\rm{S}}{{\rm{D}}}_{{\rm{B}}{\rm{l}}{\rm{o}}{\rm{o}}{\rm{d}}})}^{2}}}$$

### Statistical analysis

All statistical analyses were performed using the Statistical Package for the Social Sciences (SPSS v19.0, International Business Machines, Armonk, New York, USA). Continuous variables are presented as the mean ± standard deviation. Differences between means were tested with the paired t-test and one-way analysis of variance as appropriate. Categorical variables were analysed using the Pearson Chi-square test. Comparisons of parametric variables between the conventional technique and the real-time TPAT were performed using paired t-tests. The Wilcoxon rank-sum test was used to compare the image quality score. The agreement was tested by calculating the mean bias from Bland-Altman analysis. Categorical variables are expressed as frequencies or percentages. A p-value of less than 0.05 was considered statistically significant, and all statistical tests were 2-sided.

## Data Availability

The data used to support the findings of this study are available from the corresponding author upon request.
